# Multimodal cardiac imaging and endoscopic ultrasound-guided fine needle aspiration for accurate diagnosis and management of giant primary pericardial schwannoma: a case report with literature review

**DOI:** 10.3389/fcvm.2025.1688224

**Published:** 2025-10-30

**Authors:** Ali Hakan Konuş

**Affiliations:** Department of Cardiology, Bingöl State Hospital, Bingöl, Türkiye

**Keywords:** primary pericardial schwannoma, cardiac imaging, endoscopic ultrasound-guided fine needle aspiration, surgery, heart failure

## Abstract

**Background:**

Primary pericardial schwannoma is a highly unusual tumor, and only a few cases have been reported in the literature. We report a case of giant primary pericardial schwannoma that was accurately diagnosed and managed by multimodal cardiac imaging and transesophageal endoscopic ultrasound-guided fine needle aspiration (EUS-FNA).

**Case:**

A 47-year-old female patient presented with complaints of exertional dyspnea and non-anginal chest pain. The patient's New York Heart Association (NYHA) functional capacity score was 2–3 and N-terminus pro-B type natriuretic peptide (NT-proBNP) was elevated at 684 pg/ml. Transthoracic echocardiography (TTE) and transesophageal echocardiography (TEE) revealed a well-circumscribed mass containing a cystic lesion, compressing the left atrium (LA) and inferior vena cava (IVC). Thoracic computed tomography (CT) angiography revealed that the lesion was intrapericardial, located in the posterior mediastinum, 10.1 cm × 8.1 cm × 5.2 cm in size, had regular borders, and was compressing the esophagus. Magnetic resonance imaging (MRI) showed a well-circumscribed, T1-hypointense and T2-hyperintense pericardial mass. The mass was observed to have homogeneous signal intensity on T1 and T2-weighted images. CT and MRI showed that the mass did not cause myocardial involvement. Whole-body 18 F-fluorodeoxyglucose (18F-FDG) positron emission tomography (PET)-CT images revealed that the mass was primary, had no metastasis, and had mild to moderate 18F-FDG avidity. Immunohistochemical evaluation with EUS-FNA determined the mass to be consistent with schwannoma, and the Ki-67 index was less than 1%. The mass was completely removed after the pericardium was opened by performing a median sternotomy approach. No residual mass was detected in the patient's follow-up. At 6-month and 1-year follow-ups, there were no symptoms, the NYHA score was 1, and NT-proBNP was normal.

**Conclusion:**

The integrative approach of multimodal cardiac imaging and EUS-FNA can effectively guide the surgical approach and management of primary pericardial schwannomas preoperatively. EUS-FNA may be an effective and safe method in the management of cardiac schwannomas. To our knowledge, this is the first case in which EUS-FNA has been used for the diagnosis of cardiac schwannoma.

## Introduction

Primary pericardial tumors are highly rare neoplasms. Primary pericardial schwannoma is much rarer, and only a few cases have been reported in the literature. Due to its rarity, diagnosis and management can be challenging. Especially giant (>10 cm) forms can create a difficult clinical scenario. We report a case of giant primary pericardial schwannoma that was accurately diagnosed by multimodal cardiac imaging and transesophageal endoscopic ultrasound-guided fine needle aspiration (EUS-FNA) and successfully managed in the long term by complete surgical excision of the mass.

## Case report

**Figure 1:**
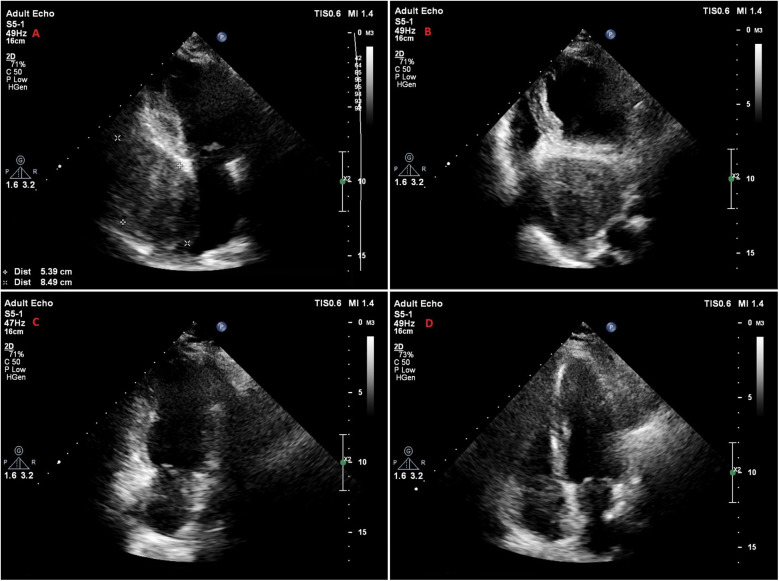
Transthoracic echocardiography images of the patient. **(A,B)** Apical 2-chamber **(A)** and apical 4-chamber **(B)** views of a hypoechoic mass located posterior to the left atrium and left ventricle, and compressing the left atrium. **(C,D)** Apical 2-chamber **(C)** and apical 4-chamber **(D)** views of the patient at one year follow-up, showing that the mass was successfully removed.

**Figure 2:**
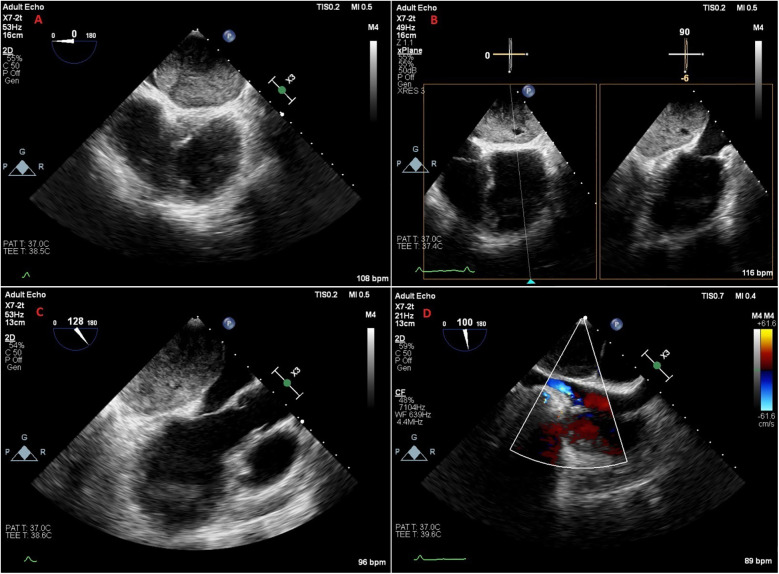
Transesophageal echocardiography images of the patient. **(A)** Midesophageal 4-chamber view. **(B)** Biplane view showing that the mass contains an area of cystic degeneration. **(C)** Midesophageal long axis view showing that the mass is compressing the left atrium, mitral annulus and partially the left ventricle. **(D)** Midesophageal bicaval view showing the mass creating mild compression on the inferior vena cava.

**Figure 3:**
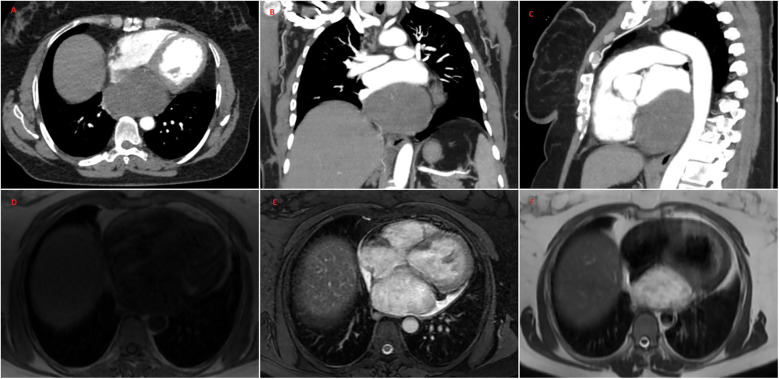
Thoracic CT angiography **(A–C)** and MRI **(D–F)** images of the patient. CT and MRI show a well-circumscribed pericardial mass measuring 10.1 cm × 8.1 cm × 5.2 cm located in the posterior mediastinum. CT and MRI demonstrated no evidence of the invasion of the inferior vena cava, left atrium, left ventricle and pulmonary veins. MRI showed that the mass was T1-hypointense **(D)** and T2-hyperintense **(F)** The mass was observed to have homogeneous signal intensity on T1 and T2-weighted images **(D–F)** The mass showed high signal intensity on T2-weighted fat suppression sequence **(E)** CT, computed tomography; MRI, magnetic resonance imaging.

**Figure 4:**
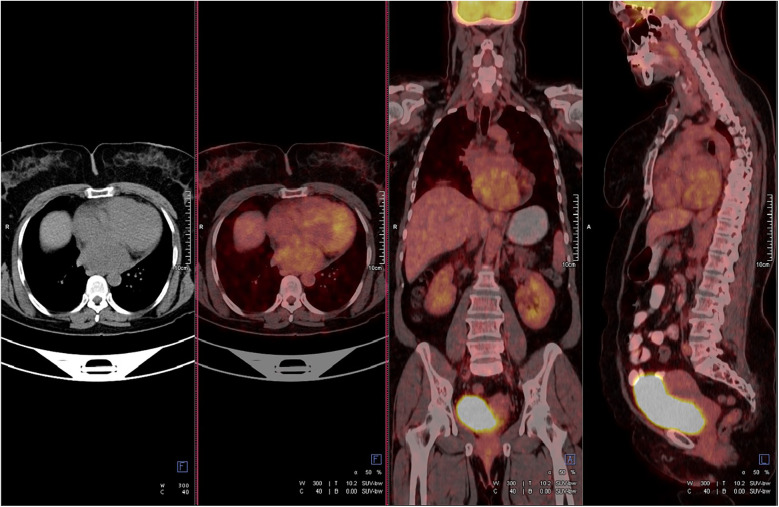
Positron emission tomography-computed tomography showed mild to moderate fluorodeoxyglucose uptake in the mass. The standard uptake value was ∼5.2. No metastatic lesions were detected.

A 47-year-old female patient applied to the cardiology outpatient clinic with complaints of exertional dyspnea and intermittent chest pain. The patient's New York Heart Association (NYHA) functional capacity score was 2–3. Chest pain was unrelated to exertion. The patient had hypertension and was normotensive under perindopril 10 mg and amlodipine 10 mg treatments. She was a nonsmoker and had Class I obesity with a body mass index of 30.8 kg/m^2^. Physical examination was normal. Laboratory parameters showed an elevation of low-density lipoprotein at 187 mg/dl and an elevation of N-terminus pro-B-type natriuretic peptide (NT-proBNP) at 684 pg/ml. The electrocardiogram showed sinus rhythm with a rate of 83 bpm and no abnormalities. Transthoracic echocardiography (TTE) revealed a well-circumscribed hypoechoic mass lesion measuring 8.5 cm × 5.4 cm, located behind the posterior walls of the left atrium and left ventricle, causing significant compression of the left atrium (Figures 1A,B). Other findings of TTE were; normal left ventricular systolic function with a left ventricular ejection fraction (LVEF) of 65%, mild dilatation of the left atrium with a left atrial volume index (LAVI) of 36 ml/m^2^, mild mitral valve regurgitation, mild tricuspid valve regurgitation, tricuspid valve regurgitation velocity 3.2 m/s, grade II diastolic dysfunction and normal right ventricular systolic function with a tricuspid annular plane systolic excursion of 22 mm. Transesophageal echocardiography (TEE) was performed for detailed evaluation of the mass and its neighbors. TEE showed a mass containing a cystic lesion that caused significant compression of the left atrium (LA) and mild compression of the inferior vena cava (IVC) (Figure 2). No pulmonary venous return anomaly or obvious compression of the pulmonary veins was detected on TEE. Thoracic computed tomography (CT) angiography revealed a pericardial mass lesion measuring 10.1 cm × 8.1 cm × 5.2 cm in size, with regular borders and soft tissue density, located in the posterior mediastinum, adjacent to the LA and descending aorta, creating significant compression on the esophagus (Figure 3). The whole-body 18F-fluorodeoxyglucose (18F-FDG) positron emission tomography (PET)-CT images revealed a giant pericardial mass with mild to moderate 18F-FDG avidity (Figure 4). No metastatic lesions were detected. Magnetic resonance imaging (MRI) showed a well circumscribed, T1-hypointense and T2-hyperintense pericardial mass. The mass was observed to have homogeneous signal intensity on T1 and T2-weighted images. There was no direct myocardial infiltration and/or left atrial invasion (Figure 3). Contrast-enhanced images were not obtained for the patient who had a history of anaphylactic shock due to a gadolinium-based contrast agent. CT and MRI showed that the mass did not cause myocardial involvement, however, the mass could not be demarcated from the LA and IVC. The coronary angiogram did not detect obstructive coronary artery disease or coronary artery compression due to the mass. For the histological diagnosis of this giant pericardial mass, EUS-FNA was performed. Histological evaluation revealed foci of spindle cell proliferation forming Antoni A and Antoni B areas. Immunohistochemical studies revealed that these spindle cells stained diffusely positive for S100, which was consistent with schwannoma. The Ki-67 index was less than 1%.

The patient was decided to undergo surgical resection by the cardiac team. The mass was revealed after the pericardium was opened by performing a median sternotomy approach with del Nido cardioplegia. The mass was found to be attached to the left atrium and inferior vena cava. With the left atrium incision, it was seen that the mass was not located under the endocardium, and the mass was easily separated from the left atrium through the incision. The inferior vena cava was also explored, and the mass was separated. The mass was completely removed, and the left atrium and inferior vena cava were repaired with polypropylene sutures. Pathological analysis of the well-circumscribed solid tissue, covered with an off-white membrane and containing hemorrhagic areas, confirmed the preoperatively detected diagnosis of schwannoma.

The patient's in-hospital follow-up was uneventful, and she was discharged on the 9th postoperative day. No mass was detected in the patient's echocardiographic follow-up (Figures 1C,D). The patient's NYHA score was 1 at 6-month follow-up, and she had no chest pain. PET-CT showed no residual lesion or metastasis. TTE performed at 1-year follow-up revealed that LVEF was 65%, LAVI (30 ml/m^2^), and diastolic functions were normal. At 1-year follow-up, NT-proBNP was normal, and the NYHA score was 1.

## Discussion

The prevalence of primary cardiac tumors has been reported as 0.02%–0.056% and they are highly rare neoplasms (1). Primary pericardial tumors are much rarer, and they have a prevalence of approximately 0.001%–0.007% (1). Schwannomas, also known as neurinoma or neurilemoma, are nerve sheath tumors that originate from Schwann cells, which produce myelin for peripheral nerves. These tumors can be benign or malignant. Benign forms are called schwannoma or benign schwannoma, while malignant forms are called malignant peripheral nerve sheath tumors or malignant schwannoma. It has been reported that 9% of all schwannomas are mediastinal, and the most common mediastinal location is the posterior mediastinum (2). Primary pericardial schwannoma is incredibly rare, with only a few cases reported in the literature (3–15). Therefore, accurate characterization of the mass with TTE, TEE, CT, and MRI imaging modalities for such rare tumors is of great importance in diagnosis and differential diagnosis, determining the surgical method, and developing management strategies.

The symptoms and clinical features of primary pericardial schwannoma are variable. Fourteen cases have been reported in the literature, including our case (Table 1); 2 cases had non-anginal chest pain, 2 case had cough and febrile or subfebrile temperature, 1 case had typical angina and acute coronary syndrome, 1 case had chest tightness, exertional dyspnea and bronchial asthma, 1 case had progressive shortness of breath and fatigue, and 1 case had dyspnea, orthopnea, substernal chest pain and heart failure. In the remaining 5 patients, the mass was detected incidentally. Our patient had intermittent non-anginal chest pain and exertional dyspnea. In our patient, TTE showed impaired diastolic dysfunction and elevated filling pressure in our patient according to current guidelines (16). Our patient, who had a NYHA score of 2–3 and elevated NT-proBNP, had heart failure with preserved ejection fraction. After mass removal, the patient's echocardiographic diastolic functions and NT-probnp were normal, and the NYHA score was 1.

**Table 1:** Characteristics of primary pericardial schwannoma cases reported in the literature.

No.	Reference	Year	Age	Sex	Initial clinic or detection	Location of tumor	Tumor size (cm)	Tumor behavior	Preoperative biopsy	Surgical approach and treatment strategy
1	(3)	1998	46	F	Incidental detection	Between the right atrium, superior vena cava, ascending aorta, and right pulmonary artery	12 × 8 × 7	Benign	No	Sternotomy with CPB
2	(4)	2000	24	M	Progressive shortness of breath and fatigue	Between the diaphragm and the diaphragmatic walls of the right atrium and right ventricle	10.1 × 4.9	Malignant	Yes (surgical biopsy)	Not reported
3	(5)	2006	42	F	Cough and subfebrile temperature	Next to the right ventricular free wall and right ventricle outflow tract	14 × 9	Benign	No	Sternotomy CPB not reported
4	(6)	2010	60	M	Resting dyspnea, orthopnea, substernal chest pain and acute heart failure	Posterior to the left atrium and left ventricle, in the posterior mediastinum	5.2 × 7 × 11	Malignant	No	Surgical method not reported Partial resection + adjuvant radiotherapy and chemotherapy
5	(7)	2013	38	F	Typical angina and acute coronary syndrome	Next to the left ventricular lateral wall and left atrium	Not reported	Benign	No	Not reported
6	(8)	2013	50	F	Cough and fever	Under the aortic arch; compressing on the right atrium, SVC and trachea; adherent to the SVC, left atrium, left hilus of lung and pulmonary artery	9 × 11	Benign	No	Thoracotomy CPB not reported
7	(9)	2014	35	M	Intermittent chest pain	Between the posterior wall of left atrium, right pulmonary artery and the carina	2.5 × 2.5 × 2	Benign	No	Median sternotomy without CPB
8	(10)	2015	42	F	Incidental detection	In the right paracardiac area between the SVC and the right upper pulmonary vein, on the right side of the ascending aorta	14 × 10 × 7	Benign	No	Sternotomy with CPB
9	(11)	2016	48	F	Chest tightness, exertional dyspnea and bronchial asthma	Next to the right atrium	2.5 × 2.6	Benign	No	Video-assisted thoracoscopic surgery
10	(12)	2018	30	M	Left chest pain	Located subcarinally and adjacent to the left atrium	3 × 2	Benign	No	Thoracotomy without CPB
11	(13)	2019	33	M	Incidental detection	On the left side of the RVOT and pulmonary artery	5 × 4	Benign	No	Thoracotomy with CPB
12	(14)	2021	70	F	Incidental detection	Posterior pericardial mass compressing the left atrium and left ventricle	4.7 × 5.9 × 8.1	Benign	Yes (CT-guided)	Thoracotomy CPB not reported
13	(15)	2025	66	M	Incidental detection	Located in the right atrioventricular groove and compressing the right atrium and right ventricle	5.6X5.3	Benign	No	Thoracotomy with CPB
Present case		2025	47	F	Intermittent non-anginal chest pain, exertional dyspnea and heart failure	Posterior to the left atrium and left ventricle, in the posterior mediastinum	10.1 × 8.1 × 5.2	Benign	Yes (EUS-FNA)	Sternotomy with CPB

SVC, superior vena cava; CPB, cardiopulmonary bypass; RVOT, right ventricular outflow tract; F, female; M, male; CT, computed tomography; EUS-FNA, endoscopic ultrasound-guided fine needle aspiration.

Multimodal cardiac imaging is essential in the diagnosis and management of pericardial masses. The first step in the diagnosis of pericardial masses is TTE. TTE can determine the localization and size of the mass, the presence of pericardial effusion, and cardiac functions. It is very useful in determining the relationship of the tumor with cardiac structures, the presence of cardiac compression, and whether it causes hemodynamic disturbances. Since it is a non-invasive diagnostic tool, it is also used in the long-term follow-up of cardiac tumors. In our case, we used TTE as a tool for the detection of a mass, determination of cardiac functions, and long-term follow-up. TEE is a useful diagnostic method for cardiac masses because it has better echogenicity and is more sensitive than TTE. It may be insufficient in the examination of masses distant from the transducer, but as in our case, it can provide much more detailed information about the characterization and anatomical adjacency of a cardiac mass located in the posterior mediastinum, which is closely related to the esophagus. Heterogeneous well-circumscribed lesions, often containing cystic degeneration areas, sometimes hemorrhage, and rarely calcification, are imaging features of cardiac schwannomas (17). As in our case, cystic changes can be more clearly identified with TEE compared to TTE. In addition, TEE can initially help evaluate the relationship of the mass with vascular structures and shed light on the surgical approach. CT can show the anatomic adjacency of the mass and invasion of surrounding tissues. It helps to reduce possible differential diagnoses after TTE and TEE, depending on the attenuation values and contrast enhancement patterns of the mass. CT detects small cardiac schwannoma masses as homogeneous enhancement, while large cardiac schwannoma masses usually show heterogeneous enhancement. PET/CT is a staging tool that evaluates whether the pericardial mass is metastatic or primary, and whether the primary lesion has spread locally or distantly. MRI provides better soft tissue characterization compared with CT. MRI has a better ability to demonstrate myocardial invasion than CT. In our case, although CT and MRI could not demarcate the pericardial mass from the LA and IVC, MRI did not detect obvious myocardial invasion. Cardiac schwannomas show T1-hypointense or isointense, T2-hyperintense signal characteristics on MRI. MRI shows heterogeneous enhancement on late gadolinium contrast imaging in cardiac schwannoma masses (14, 15, 18). Although the giant pericardial schwannoma mass in our case showed a homogeneous signal intensity on MRI, this rare condition has been reported previously (18). Contrast-enhanced MRI findings, and especially cardiac MRI parametric mapping, could have detected the heterogeneous structure of the mass in our case, which was detected by the small cystic degeneration area clearly seen on TEE. Contrast-enhanced images were not obtained for the patient who had a history of anaphylactic shock due to gadolinium-based contrast agent. In addition, cardiac MRI not only provides diagnostic evaluation of the mass but also plays a role in risk stratification (19, 20). In this context, cardiac MRI is a predictor of mortality in patients with cardiac tumors based on clinical risk factors (21). Finally, the relationship between the cardiac masses that are candidates for surgery, and the coronary arteries should be investigated preoperatively. We did not detect any relationship between the mass and the coronary arteries with conventional coronary angiography. TTE, TEE, CT, MRI, and coronary imaging are complementary diagnostic modalities in characterizing cardiac masses, guiding surgical approach, and determining treatment strategy. They are essential in improving the prognosis of patients.

Our patient had many of the typical imaging features of cardiac schwannoma (well-defined, mass containing cystic degeneration areas). After removal of the mass, pathological examination revealed hemorrhagic areas, which are sometimes seen in cardiac schwannomas. Calcification, which is rarely found in cardiac schwannomas, was not present in our case. We considered the following in the differential diagnosis of the pericardial mass in our case with imaging modalities. First, the most common non-neoplastic lesion is a pericardial cyst, which presents itself as a hypoechogenic homogeneous mass on TTE and TEE due to the fluid it contains. The signal intensities in the T1- and T2-weighted images on MRI confirmed that the mass was not a pericardial cyst. Second, the most common pericardial neoplastic masses are metastatic disease. We showed with PET/CT that the mass was primary, not metastatic. Third, lipomas contain dense fatty tissue and show a hypointense signal in T2-weighted fat suppression sequence images. For all these reasons, pericardial cyst, metastatic disease, and lipoma were not considered in our patient.

The treatment of cardiac tumors may vary according to preoperative imaging features (22). However, histopathological examination of tumor tissue is often required for definitive diagnosis and determination of whether the tumor is benign or malignant. In a case-based meta-analysis of primary cardiac schwannomas that did not include pericardial schwannomas, only 4 out of 53 cases were diagnosed with preoperative biopsy, and tumor aggressiveness was determined (23). There are a total of 14 cases in the literature about primary pericardial schwannomas, including our patient (Table 1). Only 3 cases, including our case, were diagnosed with preoperative biopsy, and the tumor type and behavior were determined. One case was diagnosed with CT-guided biopsy, the other with invasive surgical biopsy, and this case with EUS-FNA. Malignant cardiac schwannomas are not rare, and in the case-based meta-analysis, tumor behavior was malignant in more than half of the 53 patients (23). 2 out of 14 primary pericardial schwannomas reported in the literature, including our patient (benign), were malignant (Table 1). Detecting the benign or malignant behavior of primary cardiac schwannomas preoperatively is important in deciding the best individual treatment approach. The main treatment method for benign and malignant cardiac schwannomas is surgery. Chemotherapy and radiotherapy can be considered as salvage treatment in cases where the tumor is inoperable due to anatomic or patient-related factors, where the tumor has recurred and is locally advanced (24, 25). Although long-term outcomes are unknown, neoadjuvant chemotherapy and radiotherapy may be considered in locally advanced cases (24, 25). Therefore, preoperative definitive diagnosis and biopsy findings of primary cardiac schwannomas are important for determining the patient's treatment strategy and improving the patient's prognosis.

The methods currently used for the definitive diagnosis of mediastinal lesions are: open thoracic biopsy under mediastinoscopy, ultrasound or CT-guided biopsy, bronchoscopy-guided fine needle aspiration, and EUS-FNA. The advantages of EUS-FNA compared with other methods are high sensitivity and specificity, easy access to posterior mediastinal lesions, easy and uncomplex procedure, low risk, few complications, and low cost (26–28). Other methods mentioned above, except EUS-FNA, may cause trauma, respiratory failure, and even cardiac arrest. EUS-FNA is considered a relatively safe, rapid, easily reproducible, and minimally invasive technique compared to other methods. In a study including 107 patients, the sensitivity, specificity, positive predictive value, and negative predictive value of EUS-FNA for the diagnosis of mediastinal lesions were reported to be 92%, 100%, 100%, and 85%, respectively (29). No obvious complications were observed in the patients (29). All this evidence demonstrates the potential value of EUS-FNA in the diagnosis of mediastinal masses. EUS-FNA is not capable of accessing all mediastinal and cardiac masses. EUS-FNA is a suitable tool for the diagnosis of subcarinal and posterior mediastinal masses (30). EUS-FNA is an alternative method that may also allow the diagnosis of mediastinal masses superior to the aortic arch (31). It can also be a safe and effective method for the diagnosis of pericardial masses, whether or not they have myocardial invasion (32). EUS-FNA has limitations for pericardial masses located in the anterior mediastinum and other pericardial masses for which transesophageal access is not possible. The efficacy and safety of EUS-FNA in the diagnosis of intracardiac masses are unknown. Although case-by-case diagnostic success with EUS-FNA for left atrial masses has been reported in the literature (33), EUS-FNA is generally not considered a suitable method for the diagnosis of intracardiac masses. In our case, there was a posterior mediastinal mass, and we quickly and reliably diagnosed a benign schwannoma preoperatively by EUS-FNA. This diagnosis was confirmed by pathological examination of the mass after surgery. To our knowledge, this is the first case of primary cardiac schwannoma diagnosed by EUS-FNA.

The decision regarding the surgical approach and whether to use cardiopulmonary bypass (CPB) is determined based on the imaging features of the tumor, such as its location, size, relationship to surrounding tissues, and presence of myocardial infiltration, as well as the clinical condition and comorbidities of the patient. Median sternotomy and cardiac arrest with CPB is the most frequently used method in the literature for benign or malignant primary intracardiac tumors (34). In parallel with this, in the literature, median sternotomy and cardiac arrest with CPB has been the most commonly used surgical approach for the resection of benign or malignant primary intracardiac schwannomas, and less invasive surgical methods have rarely been used (35). Unlike primary intracardiac tumors, primary pericardial tumors may allow for a less invasive surgical approach. Therefore, surgical resection with thoracotomy is the main method for benign pericardial tumors. Although the thoracotomy approach has advantages over sternotomy in terms of preventing mediastinitis, it is technically more difficult due to the limited operating area. However, the adhesion and invasiveness of the tumor make sternotomy superior in surgical approach. Median sternotomy and cardiac arrest with CPB is the preferred method for pericardial tumors with myocardial infiltration or located posterior to the heart. In our case, we preferred the median sternotomy and cardiac arrest with CPB method because the giant benign primary pericardial schwannoma mass was located in the posterior mediastinum and appeared to have adhesion to the LA and IVC. Including our case in the literature, the surgical approach has been reported in 11 of 14 primary pericardial schwannoma masses (Table 1). Of these, sternotomy with or without CPB was used in 5 cases, and thoracotomy with or without CPB was used in 5 cases. In 1 case, it was reported that no obvious adhesion or invasion to surrounding tissues was detected on imaging, allowing complete tumor resection with video-assisted thoracoscopic surgery, which is a less invasive approach than sternotomy and thoracotomy. In the preoperative period, detailed imaging evaluation and histopathological determination of tumor behavior play a key role in the complete removal of primary cardiac schwannomas using invasive or minimally invasive methods.

## Conclusion

Primary pericardial schwannomas are exceedingly rare. A comprehensive evaluation of the mass with TTE, TEE, CT, and MRI may help improve the prognosis of patients. According to imaging findings, a well-circumscribed solid pericardial mass with cystic degeneration located in the posterior mediastinum should bring schwannoma into consideration in the differential diagnosis. This case shows that EUS-FNA can be an effective and reliable method for the diagnosis of cardiac schwannomas. To our knowledge, this is the first case in which EUS-FNA has been used for the diagnosis of cardiac schwannoma. The integrative approach, including comprehensive cardiac imaging and EUS-FNA in deciding on invasive or less invasive surgical approaches and optimal treatment strategies, yielded a successful outcome in this case.

## Data Availability

The original contributions presented in the study are included in the article/Supplementary Material, further inquiries can be directed to the corresponding author.
